# Shall the Radcliffe Infirmary Be Rebuilt?

**Published:** 1891-05-30

**Authors:** 


### Shall the Radcliffe Infirmary be Rebuilt?

The following appeared in the *Oxford Chronicle* last week:—

We reprint in full elsewhere an article taken from The Hospital, the title being "Oxford on its Mettle." The concluding words of the article which we quoted last week are: "Oxford must build itself a new hospital. The honour of the county, of the city, and of the University alike demands it. We hope that, notwithstanding any little sensitiveness which our criticisms may have aroused, we shall not be forbidden the honour of a share in the erection of the new buildings." "Can this mean," we asked, "that The Hospital is coming down handsomely in the event of the work alluded to being carried out?" Our query, we are glad to say, has produced a very practical response. We are now authorised to say that the proprietors of The Hospital, feeling strongly that our greatest University should have a hospital of a most complete and perfect character, are prepared to give a subscription of one hundred guineas provided it is decided to rebuild the older portions of the Infirmary and to form a committee to raise the £10,000 required to carry out the necessary works. The Hospital is further prepared to throw open its columns to advocating this rebuilding, and to use its influence to help the committee in raising the required funds. We would ask the Infirmary authorities seriously to consider whether the time has not arrived when they might make a determined effort to accomplish in a thorough manner the rebuilding of the hospital. If the bull were taken by the horns we feel certain that in such a noble undertaking the city and county would rally thoroughly round them, and Oxford would take a worthy place in regard to its hospital accommodation. The example set by Derby is a splendid one, and if a start were once made Oxonians, we feel convinced, would spend themselves and be spent in attaining such a desirable consummation as that which the Derby people have achieved.

